# Comprehensive virome analysis of the viral spectrum in paediatric patients diagnosed with *Mycoplasma pneumoniae* pneumonia

**DOI:** 10.1186/s12985-022-01914-y

**Published:** 2022-11-09

**Authors:** Qiong Guo, Lili Li, Chao Wang, Yiman Huang, Fenlian Ma, Shanshan Cong, Jingjing Tan, Lihong Yao, Aijun Chen, Lishu Zheng

**Affiliations:** 1 NHC Key Laboratory of Medical Virology and Viral Diseases, National Institute for Viral Disease Control and Prevention, China CDC, Beijing, China; 2 Institute for HIV/AIDS and STD Prevention and Control, Beijing Center for Disease Prevention and Control, Beijing, China

**Keywords:** Next-generation sequencing, Pathogen spectrum, Metagenomics analysis, Respiratory virus, *Mycoplasma pneumoniae*

## Abstract

**Background:**

Among hospitalized children suffering from community-acquired pneumonia, *Mycoplasma pneumoniae* (MP) is one of the most common pathogens. MP often exists as a co-infection with bacteria or viruses, which can exacerbate the clinical symptoms. We investigated the pathogen spectrum in MP-positive and MP-negative samples from hospitalized children with respiratory tract infections in Beijing, China.

**Method:**

This study included 1038 samples of nasopharyngeal aspirates obtained between April, 2017 and March, 2018 from hospitalized children under 6 years of age with respiratory tract infections. To explore the impact of MP infection on the composition of the pathogen spectrum, 185 nasopharyngeal aspirates (83 MP-positive/102 MP-negative) were randomly selected for next-generation sequencing and comprehensive metagenomics analysis. Real-time PCR was used to detect and verify common respiratory viruses.

**Results:**

Of the 1038 samples, 454 (43.7%) were infected with MP. In children < 6 years of age, the MP infection rate gradually increased with age, with the highest rate of 74.2% in 5–6-year-olds. The results of metagenomics analysis revealed 11 human, animal and plant virus families, and bacteriophages, including common respiratory viruses, enteroviruses and anelloviruses. The virus family with the highest number of reads in both MP-positive and MP-negative samples was the *Pneumoviridae*, and the number of reads for human respiratory syncytial virus (HRSV) in MP-positive samples was higher than that in MP-negative samples. Among the 83 MP-positive samples, 47 (56.63%) were co-infected with viruses, the most common of which was influenza virus (IFV). The durations of hospitalization and fever were higher in patients with MP co-infection than MP single infection, but the difference was not statistically significant.

**Conclusion:**

The viral family with the highest number of reads in both groups was *Pneumoviridae*, and the number of reads matched to HRSV in MP-positive samples was much higher than MP-negative samples. Co-infection of MP and IFV infection were the most cases.

**Supplementary Information:**

The online version contains supplementary material available at 10.1186/s12985-022-01914-y.

## Background

*Mycoplasma pneumoniae* (MP) is a pathogenic microorganism that contains DNA and RNA and lacks a cell wall. It is a common pathogen that causes respiratory tract infections (RTIs). *Mycoplasma pneumoniae* pneumonia (MPP) is a major cause of community-acquired pneumonia in hospitalized children [1]. It is estimated that MPP is responsible for approximately 3–10% of respiratory infections in children [2], and approximately 5–12% of the hospitalized children with MPP were admitted to the intensive care unit [3–5]. The clinical presentation of MPP is usually tracheobronchitis, pneumonia, nonspecific symptoms of upper RTI (such as headache, sore throat, rhinitis, otitis media) and extrapulmonary manifestations (such as encephalitis, urticaria, allergic purpura) [6]. MPP is generally considered a mild and self-limiting disease, but in some cases can lead to sever pneumonia, which can be life-threatening in children [7]. It is reported that MP can damage human airway epithelial cells and cilia, and can affect the function of the mucus-ciliary clearance system and host immune function [8]. Additionally, recent research showed that indirect (immune-mediated) mechanisms are mainly implicated in* Mycoplasma pneumoniae*-related extra-pulmonary diseases (MpEPDs), such as Henoch-Schonlein purpura, Myositis, Kawasaki disease, Nephritis [9]. A cohort study had found that incident cases of early-onset and late-onset asthma might be related to MPP [10]. MP occurs worldwide and is easily transmitted by direct respiratory droplet contact. MPP can cause infections in people of any age, and is one of the leading causes of community-acquired pneumonia (CAP), especially in children [5, 11].

In addition to MP, respiratory viruses also cause RTIs in children. Human respiratory syncytial virus (HRSV), human adenovirus (HAdV) and influenza virus (IFV) are the most common respiratory viruses infecting infants and children under 5 years of age [12]. In children with lower RTIs, approximately 31–66% of cases are caused by co-infection of respiratory viruses and bacteria [13]. In recent years, it has been reported that MP can co-infect a host along with bacteria and viruses (such as *Streptococcus pneumoniae*, HRSV) and the co-infection rate can reach 52% [14]. Compared with mono-infection, MP co-infection cause serious clinical symptoms and the clinical manifestations are more diverse [7]. The relationship between MP and viruses is yet to be fully elucidated.

Metagenomic next generation sequencing (NGS) can simultaneously detect multiple viruses, as well as new and highly-differentiated viruses, providing comprehensive detection and quantitative analysis of all microorganisms present in clinical samples [15]. In this study, to analyze the pathogenic spectrum of MP-positive and MP-negative samples in hospitalized children with RTIs, nasopharyngeal aspirate (NPA) samples were collected and sequenced using NGS technology. By analyzing the NGS sequences of the two groups, we investigated the impact of MP infection on the composition of the respiratory virus spectrum, and explore whether MP infection affect the diversity and complexity of virus co-infection.

## Methods

### Participants and sample collection

A total of 1038 NPA samples were collected from patients under 6 years of age diagnosed with a RTIs in the pediatric inpatient unit of Beijing Friendship Hospital between April, 2017 and March, 2018. In this study, we described the epidemiological characteristics of MP infection, compared the pathogen spectrum and clinical manifestation between MP-positive group and MP-negative group. The protocol was approved by the Ethical Committee (IVDC2017-021, approval date: Mar 20, 2017). Informed consents were obtained by the legal guardians. In total, 185 samples of NPA were randomly selected using the RAND function of Excel for NGS, including 83 MP-positive samples and 102 MP-negative samples. MPP was diagnosed according to the following criteria: clinical symptoms of a cough and fever; wet rales on auscultation or infiltrative inflammatory manifestations on a chest radiograph; ≥ four-fold antibody titers increase of paired sera; a positive IgM antibody test; PCR-positive for MP; or isolation of MP from cultures of respiratory specimens [16, 17]. In the present study, Serum MP antibody titers were detected with SERODIA-MYCO II kit (Fujirebio, Japan), and titer ≥ 1:80 was considered positive.

NPAs samples were collected by professional personnel and stored in virus transport solution in an ice box during transport to the laboratory. Samples were then stored at ˗80°℃ for later use.

### Sample pretreatment and viral nucleic acid extraction

NPA samples in solution (800 µl) were centrifuged at 3000 rpm for 20 min, then 20,000 rpm for 15 min. The supernatant was filtered through a 0.22 μm filter to remove eukaryotic and bacterial cell-sized particles. The filtered sample was subjected to nuclease treatment at 37°℃ for 2 h to eliminate the free and unprotected nucleic acids, including benzonase nuclease (Novagen, Germany), TURBO™DNase (Thermo Fisher Scientific, USA) and RNaseI (Thermo Fisher Scientific). Viral nucleic acids were extracted using the QIAamp MinElute Virus Spin Kit (Qiagen, Germany) in accordance with the manufacturer’s instructions.

### cDNA library construction and high throughput sequencing

First-strand cDNA synthesis was performed using the SuperScript III First-Strand Synthesis System (Invitrogen, USA). Subsequently, double-stranded cDNA (ds-cDNA) was synthesized using 3ʹ–5ʹ exo-Klenow fragment (Biolabs, New England). The obtained ds-cDNA was amplified using the QuantiTect Whole Transcriptome Kit (Qiagen, Germany). The amplification products were sent to Beijing Liuhe Huada Gene Technology Company for NGS. The Illumina HiSeq2000 platform was used for NGS. A total of 8G clean data were obtained for each sample.

### Pathogen verification

Real-time PCR was performed on the NPA samples to detect common respiratory virus, including HAdV, HRSV, IFV (A/B/C), human parainfluenza virus (HPIV1–4), human metapneumovirus (HMPV), human polyomavirus (HPyV 3/4), human rhinovirus (HRV), human bocavirus (HBoV), and human coronavirus (HCoV 229E/OC43/NL63/ HKU1). Samples tested positive with a cycle threshold (CT) value < 37 (primers and probes are shown in T Additional file 1: Table S2). DNA viruses were detected with the TaqMan™ Gene Expression Master Mix kit (Thermo Fisher Scientific, USA) and RNA viruses were detected with the AgPath-ID™ One-Step RT-PCR kit (Ambion, USA) in accordance with the corresponding manufacture’s protocols.

### Bioinformatics analysis

The obtained sequences were processed and analyzed as follows. Prinseq-lite software was used to filter out low-quality data (QC cut-offs was listed in Additional file 1: Table S1). Then Bowtie2 alignment was performed to remove the sequences of the host, and the remaining reads were assembled to obtain contig sequences using MIRA with default parameters [15]. Blastn and Blastx were used to align the obtained reads at the nucleotide and amino acid levels against the virus reference database (RefSeq) download from Genbank, respectively, to determine the virus species and coverage. The E value was set to 1 × 10^− 5^ for blastn and blastx to maintain high sensitivity and low false-positive rate.

### Statistical analysis

SAS 9.4 software was used for statistical analysis of the data. We compared the basic information of children hospitalized with and without MP. Additionally, the clinical symptoms of MP and virus co-infection versus single MP infection were also assessed. Categorical data, such as age and gender, were tested using Pearson’s chi-square test. However, continuous variables including pneumonia diagnosis cases, white blood cell count, lymphocyte percentage, hospital stay, fever duration and max temperature were analyzed using independent-samples two-tailed t-test. Since the C-reactive protein data is non-normal, it was analyzed using Wilcoxon’s rank sum test. *P* < 0.05 was considered to indicate a statistically significant difference.

## Results

### Epidemiological characteristics of MP-positive and MP-negative samples

**Fig. 1:**
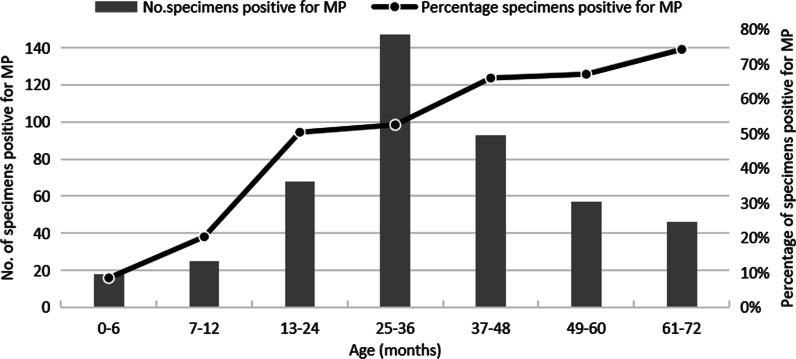
Age distribution of RTI hospitalized children infected with MP.

**Fig. 2:**
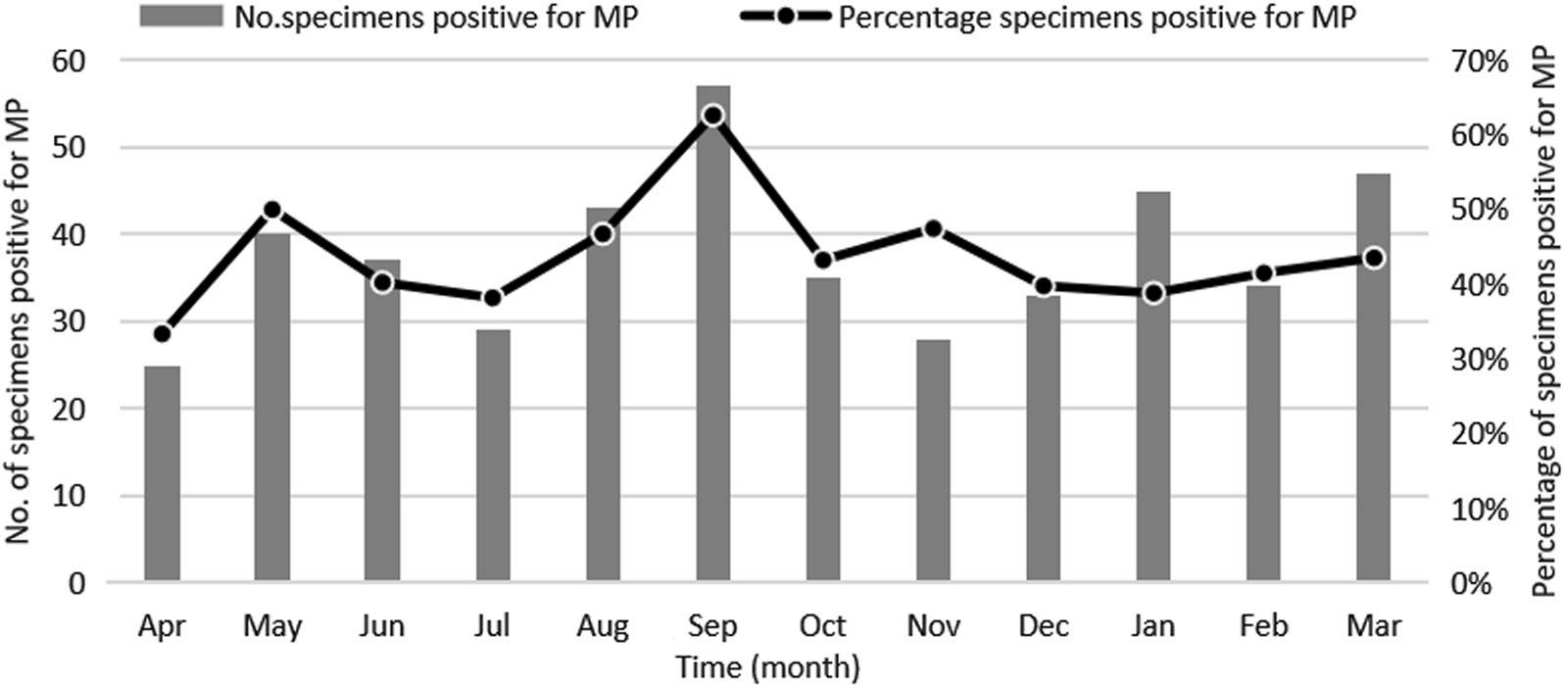
Seasonal distribution of RTI hospitalized children infected with MP.

From April, 2017 to March, 2018, a total of 1,038 NPA samples from hospitalized children, < 6 years of age, with RTIs were collected. The age distribution was 1 day to 6 years old, and the average age was 29 months. Of the total cases, 454 were positive for MP and 584 were negative. The MP infection rate was 43.7%. 953 patients were diagnosed with pneumonia in 1038 cases, and 96.7% (439/454) of MP-positive patients were diagnosed with pneumonia. As the age of children increased, the MP infection rate gradually increased, with the highest rate of 74.2% (46/62) detected among those aged 5–6 years (Fig. 1). Among the 454 MP-positive cases, most of the children were older than 2 years (75.5%, 343/454). Among the 584 MP-negative children, 359 cases (61.5%, 359/584) were under 2 years. The difference in age distribution between the two groups was statistically significant (*P* < 0.01). In the MP-positive group, males accounted for 48.7% and females accounted for 51.3% (sex ratio: 1:1.05). In the MP-negative group, 58.9% cases were from males, 41.1% were from females (sex ratio: 1.43:1) The difference in sex ratio between the two groups was statistically significant (*P* < 0.01) (Table 1). The laboratory information including white blood cell (WBC) count, lymphocyte percentage (LY%) and C-reactive protein (CRP) were also collected, among which, LY% and CRP were different between MP-positive and MP-negative groups, as shown in Table 1. Seasonal distribution results showed that the rate of MP infection in September was the highest (62.6%, 57/91), but there was no obvious seasonal epidemic (Fig. 2). Additionally, common complications in all patients were secondary thrombocythemia (562/1038), abnormal electrocardiography (227/1038), electrolyte disturbances (225/1038), lymphadenopathy (100/1038) and urticarial (12/1038). The severe complications were respiratory failure (52/1038) and heart failure (15/1038).

**Table 1:** Basic information of 1038 hospitalized children

Characters	Case No. (%)			χ^2^	*P*
	MP (+)	MP-free (-)			
*Age (month)*					
0–6	18 (4.0)	194 (33.2)		216.03	< 0.01^a^
7–12	25 (5.5)	98 (16.8)			
13–24	68 (15.0)	67 (11.5)			
25–36	147( 32.4)	133 (22.8)			
37–48	93 (20.5)	48 (8.2)			
49–60	57 (12.6)	28 (4.8)			
61–72	46 (10.1)	16 (2.7)			
*Gender*					
Male	221 (48.7)	344 (58.9)		10.77	< 0.01^a^
Female	233 (51.3)	240 (41.1)			
*Diagnosis and laboratory information*					
Pneumonia	439 (96.7)	514 (88.0)		25.61	< 0.01^b^
White blood cell count (×10^9^/L)	10.11 ± 4.44	10.71 ± 5.36		1.94	0.052^b^
Lymphocyte percentage (%)	33.99 ± 15.78	49.40 ± 20.33		13.33	< 0.01^b^
C-reactive protein (mg/L)	12.58 ± 19.67	10.21 ± 22.80		6.96	< 0.01^c^

^a^Chi-squared test

^b^Independent-samples t- test

^c^ Wilcoxon’s rank sum test

### NGS data

After sequencing 185 randomly-selected NPA samples, the obtained data were processed to removed irrelevant sequences such as host and bacterial sequences, and then compared with the NCBI virus database. The total number of reads for samples from the MP-positive group ranged from 6538 to 666,756 (median 19,811), with a mean read length of 72–130 bp, and the number of contigs ranged from 230 to 8,745 (median 703). The total number of reads for samples from the MP-negative group ranged from 4281 to 438,405 (median 15,006), with a mean read length of 57–194 bp, and the number of contigs ranged from 183 to 3341 (median 531). These reads matched to diverse viruses including human-related viruses, insect viruses, mammalian viruses, fungal viruses, phages and other viruses.

### Analysis of the pathogen spectrum

In this study, we separately analyzed torque teno virus (TTV) and other common human-related viruses. Viral spectrum analysis was performed on the common human-related viruses after removing TTV (Additional file 1: Table S3).

**Fig. 3:**
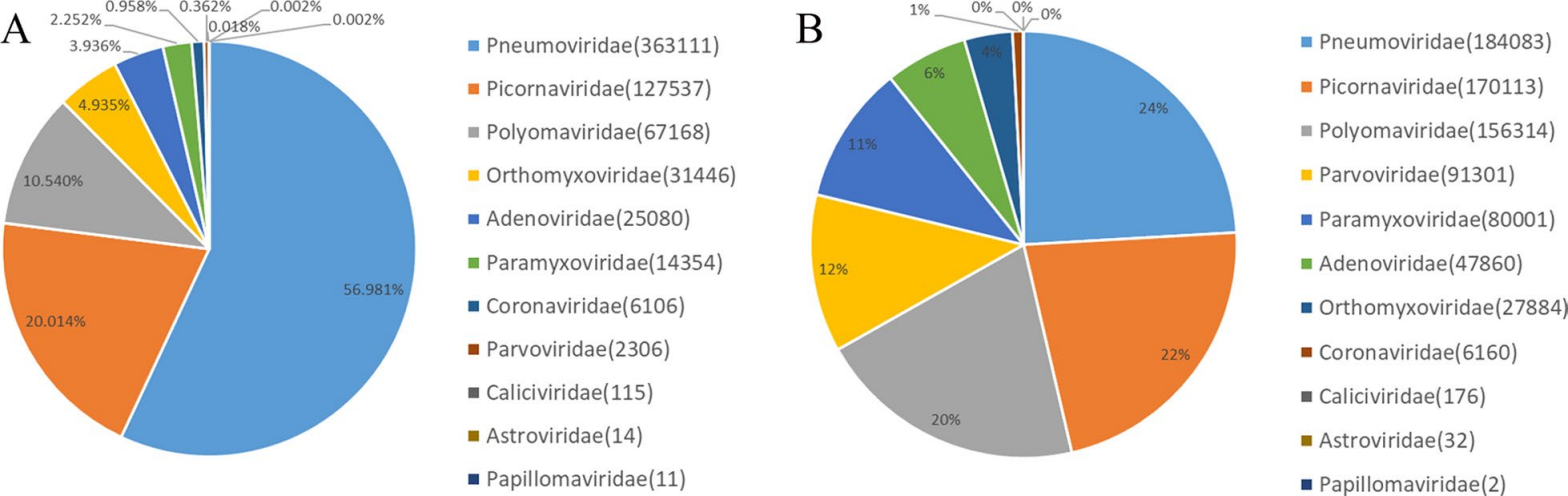
Distribution of different viral families in MP positive and negative groups. The reads of 11 viral families were sequenced by NGS. **A** MP-positive group; **B** MP-negative group

A variety of common virus-related sequences were detected in the respiratory tract samples of the MP-positive group. The total number of reads was 637,248. These virus sequences were from the following 11 viral families (Fig. 3): *Pneumoviridae*, *Picornaviridae*, *Polyomaviridae*, *Orthomyxoviridae*, *Adenoviridae*, *Paramyxoviridae*, *Coronaviridae*, *Parvoviridae*, *Caliciviridae*, *Astroviridae* and *Papillomaviridae*. Among them, reads from *Pneumoviridae*, *Picornaviridae* and *Polyomaviridae* were more abundant, with relative abundances of 56.98%, 20.01% and 10.54%, respectively. Furthermore, the virus-related sequence reads were mainly consisted of HRSV (56.98%), HRV (19.78%), HPyV (10.54%), IFV (4.93%), HAdV (3.94%) and HPIV (1.42%). Other viruses were also detected, but the number of related reads was relatively small, such as for norovirus, mamastrovirus, human rubulavirus and human papillomavirus.

In the MP-negative group, a variety of common virus-related sequences were also detected. The total number of reads was 807,989. These virus sequences were from the following 11 viral families: *Pneumoviridae*, *Picornaviridae*, *Polyomaviridae*, *Parvoviridae*, *Paramyxoviridae*, *Adenoviridae*, *Orthomyxoviridae*, *Coronaviridae*, *Caliciviridae*, *Astroviridae* and *Papillomaviridae* (Fig. 3). Different from the MP-positive group, reads from *Picornaviridae*, *Polyomaviridae*, *Parvoviridae* and *Paramyxoviridae* were more abundant, with relative abundances of 22.27% (*P* < 0.001), 20.46% (*P* < 0.001), 11.95% (*P* < 0.001) and 10.47% (*P* < 0.001), respectively. Further analysis of the viruses showed that HRV (22.04%), HRSV (24.07%), HPyV (20.46%), HBoV (11.94%), human respirovirus (10.45%), HAdV (6.27%) and IFV (3.65%) accounted for the majority of the sequencing data from MP-negative samples. Other viruses were also detected, but the number of related reads was relatively small, such as for human rubulavirus, human papillomavirus and foot-and-mouth disease virus.

The composition of the pathogen spectrum was compared between the two groups. The virus families with the highest representation among the two groups were the same, both *Pneumoviridae* and *Picornaviridae*. The proportion of *Pneumoviridae*-related virus reads among the total number of reads in the MP-positive group was 56.98%, which was much higher than that in the MP-negative group (24.10%) (*P* < 0.001). The proportion of *Polyomaviridae*, *Paramyxoviridae* and *Parvoviridae* virus-related sequences in the MP-negative group was higher than that in the MP-positive group (*P* < 0.001). Regarding specific viruses, the proportion of HRSV-related reads in the MP-positive samples was 57.0%, which was much higher than that in the MP-negative samples (24.1%) (*P* < 0.001).

### Common respiratory viruses

**Fig. 4:**
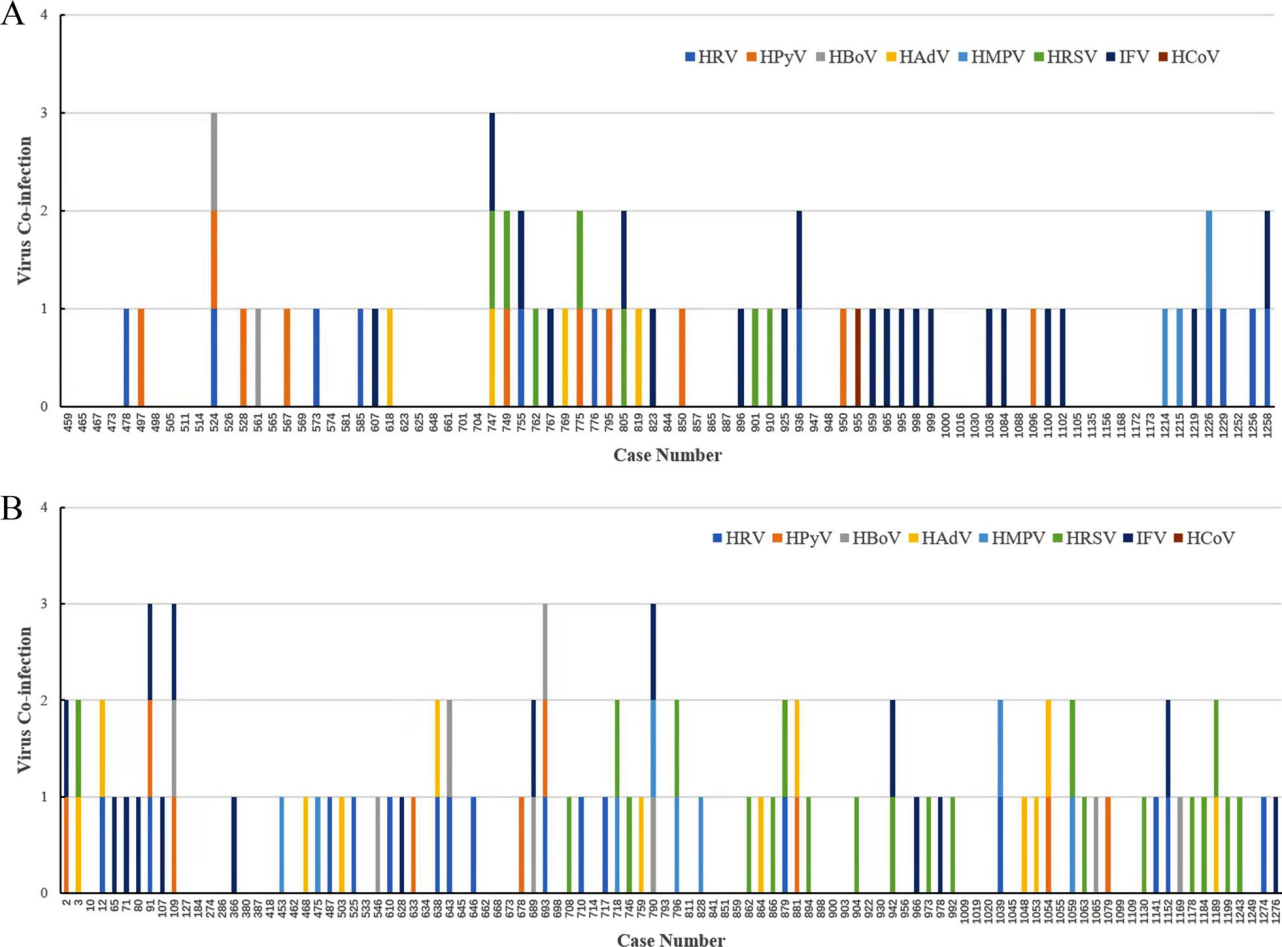
Common respiratory virus co-infection of all samples sequenced by NGS and real-time PCR. **A** 83 MP-positive cases; **B** 102 MP-negative cases. HAdV (human adenoviru), HRSV (Human respiratory syncytial virus), IFV (influenza virus A/B/C), HPIV (human parainfluenza virus 1–4), HMPV (human metapneumovirus), HPyV (human polyomavirus 3/4), HRV (human rhinovirus), HBoV (human bocavirus), and HCoV (human coronavirus, 229E/OC43/NL63/HKU1). Samples tested positive with a cycle threshold (CT) value < 37

The positive viral infection samples detected by NGS was confirmed by real-time PCR and a number of respiratory viruses were detected including HRSV, HMPV, HRV, HPyV, IFV, HAdV, HCoV and HBoV (Fig. 4 and Table 2). Among 83 cases in the MP-positive group, 47 (56.62%) were mixed infections, of which 38 (45.78%) were single virus infections, 7 (8.43%) were double virus infections, and 2 (2.41%) were triple virus infections. MP and IFV co-infection had the highest detection rate (24.10%, 20/83), followed by MP and HRSV co-infection (12.05%, 10/83). Among 102 MP-negative cases (Fig. 4), 66 (64.71%) were infected with virus. HRSV was the most commonly detected virus (20.59%, 21/102), followed by IFV (15.69%, 16/102) and HRV (15.69%, 16/102).

**Table 2:** MP co-infection with other respiratory viruses detected by real-time PCR

Groups	Co-infection viruses	No. (%)
1viruse (n = 38)	MP + HRV	11 (13.25)
	MP + HRSV	10 (12.05)
	MP + HBoV	2 (2.41)
	MP + HAdV	4 (4.82)
	MP + HMPV	3 (3.61)
	MP + HPyV	7 (8.43)
	MP + IFV	20 (24.1)
	MP + HCoV	1 (1.20)
2 viruses (n = 7)	MP + IFV + HRV	3 (3.61)
	MP + HRSV + HPyV	2 (2.41)
	MP + IFV + HRSV	1 (1.20)
	MP + HMPV + HRV	1 (1.20)
3 viruses (n = 2)	MP + HRV + HPyV + HBoV	1 (1.20)
	MP + HAdV + IFV + HCoV	1 (1.20)

### Comparison of the clinical symptoms for MP co-infection with single MP infection

To compare the clinical symptoms of MP virus co-infection with single MP infection, the clinical information for patients from the two groups was compared (Table 3). All NPAs were detected by real-time PCR to verify co-infection with common respiratory virus. The results showed that the mean number of days of hospitalization were 9.64 ± 3.87 and 8.72 ± 2.02 days for MP co-infection and MP single infection patients, respectively, but this difference was not significant (*P* > 0.05). The fever duration for patients with MP co-infection (9.74 ± 3.61 days) was similar to that of single infection patients (9.44 ± 3.08 days) (*P* > 0.05). Serum Creatine Kinase MB Isoenzyme (CK-MB) levels and the white blood cell count were approximate between MP co-infection and mono-infection patients, no significant differences were observed (*P* > 0.05).

**Table 3:** Comparison of clinical symptoms of hospitalized children infected with MP

Characteristics	With virus coinfection (n = 47)	Without virus coinfection (n = 36)	*P*
*Age (years), No. (%) *			
< 2	8 (17.0)	6(16.7)	0.75^a^
2–5	11 (23.4)	11(30.6)	
> 5	28 (59.6)	19(52.8)	
*Gender, No. (%) *			
Male	19 (40.4)	22 (61.1)	0.08^a^
Female	28 (59.6)	14 (38.9)	
Hospital stay (day)	9.64 ± 3.87	8.72 ± 2.02	0.17^b^
*Clinical presentation *			
Fever duration (day)	9.74 ± 3.61	9.44 ± 3.08	0.68^b^
Max temperature (℃)	39.61 ± 0.50	39.63 ± 0.40	0.87^b^
*Laboratory tests *			
White blood cell count (× 10^9^/L)	9.45 ± 4.07	10.04 ± 4.25	0.53^b^
Lymphocyte percentage (%)	34.54 ± 16.33	36.30 ± 14.46	0.60^b^
C-reactive protein (mg/L)	18.39 ± 21.46	17.48 ± 18.72	0.85^c^
Elevated serum CK-MB, No. (%)	11 (23.4)	6 (16.7)	0.58^a^

^a^Chi-squared test

^b^Independent-samples t- test

^c^ Wilcoxon’s rank sum test

### Anelloviruses and phage

In this study, a large number of sequence reads (34.7%) matched to the *Anelloviridae* family, mainly TTV, torque teno mini virus (TTMV), torque teno midi virus (TTMDV) and small anellovirus (SAV).

In addition to the common respiratory viruses and anelloviruses, a large number of phage-related sequences were detected, such as Bacillus phage, Escherichia phage and Enterobacter phage. Since bacteriophage are viruses that infect bacteria, the presence of phage indirectly reflects bacterial infection.

## Discussion

Respiratory infections are considered a major health threat to infants and young children [18]. MP is one of the most commonly detected bacteria responsible for mild to severe lower RTIs among older children [19]. Some respiratory viruses can also cause pneumonia, such as IFV, HRSV, HAdV, HBoV and HRV [12]. A previous study showed that in cases of MPP, co-infection with viruses and bacteria is common and the co-infection rate can reach 52% [14]. Compared with mono-infection, the symptoms associated with co-infection can be relatively more serious [20]. This study collected NPA samples between April, 2017 and March, 2018 from hospitalized children under 6 years of age with RTIs in Beijing, China. NGS and real-time PCR were used to detect the causative agents of the RTIs in the children. We compared the virus spectrum and identified the common infecting respiratory viruses between the MP-positive and MP-negative groups, found that both *Pneumoviridae* and *Picornaviridae* are the largest proportion among the two groups. 113 of the 185 NPA samples were co-infected with respiratory viruses. By contrast with MP isolated infection children, the clinical symptoms of children with MP combined with virus infection had no statistically difference.

Accurately and promptly detecting respiratory viruses is a key step prior to initiating antiviral treatment [21]. There are noticeable differences in the detection rates of viral pathogens as a result of the method used for detection. NGS is used for detecting pathogens in samples containing a mixture of different species, without the need for sequence-specific amplification [22]. The advantages of NGS include the unbiased, sensitive and simultaneous detection of multiple viral pathogens. In our study, we employed NGS to obtain comprehensive and reliable data. The results were verified by real-time PCR, and 113/185 samples (61.1%) were positive for common respiratory viruses. These data were consistent with those of a previous study that reported a 63% detection rate among samples using a PCR method [23].

RTIs present a major threat to the health of infants and young children [18], and MP is one of the most common pathogens causing mild to severe lower RTIs in children [19]. It was reported that 70.9% of MP infections occurred in children from 1 to 6 years of age in South Korea [24]. In China, 80% of MP infections occurred in children under 7 years of age [25]. In the current study of children aged under 6 years, the proportion of MP infection increased with age, peaking among children aged 5–6 years. A study in the United States showed that 73.3% of children hospitalized with MP infection were over 5 years of age [26]. This may be because younger children are less exposed to the general population, thereby reducing the risk of MP infection. In addition, the results of the current study showed that MP infection could occur throughout the year, with no obvious epidemic seasonality. However, the MP infection rate in fall was slightly higher than that in the other seasons, which was consistent with previous reports that MP infection is more common in the summer or early fall, but may occur at any time of the year [1].

Previous studies have shown that some pediatric patients with RTIs are infected with multiple respiratory pathogens at the same time. MP co-infection with bacteria and viruses is common. For example, MP and IFV, MP and HPIV, and MP and *Legionella pneumophila* all have high co-detection rates [2]. In our study, the ratio of co-infection with multiple pathogens in MP-positive cases was 56.63%, which was higher than previous reports. In MP-positive cases, the co-infection rate with IFV was highest among all cases (24.1%) and this was consistent with the findings of Zheng, et al. [27] and Kalenahalli, et al. [28]. It has been reported that viral infection can make patients susceptible to MP infection, and that this interaction may lead to the aggravation of asthma [29]. It may be that MP infection can cause cellular and humoral immunity dysfunction and induce immunosuppression in the body, damaging the patient’s systemic and local defense system, and thereby promoting virus infection [30]. These findings suggest that clinicians should consider the co-infection of virus and MP when making clinical diagnosis, and initiate treatment accordingly.

Compared with single infection with MP, the clinical symptoms in co-infected children are relatively more severe and more diverse [20]. MP and virus co-infection can prolong the clearance time of pathogenic bacteria, disrupt the host’s immune response, and prolong the inflammatory response time [8]. The results of this study showed that compared with children with MP single infection, children with MP combined with viral infection had longer hospital stays and longer fever duration, but these differences were not statistically significant. Chiu et al. compared the clinical manifestations of MP single infection, MP combined with *Streptococcus pneumoniae* infection, and MP combined with virus infection. They found that the duration of fever in children with MP and *S. pneumoniae* co-infection was longer than that of children with MP single infection, but similar to our study, there was no significant difference [31]. However, in another study, the duration of fever in children with MP combined with HAdV infection was significantly longer than for MP single infection [8]. These differences between studies may reflect the different pathogens analyzed, and the impact of particular virus on MP infection outcomes and the clinical manifestations of MPP require further investigation.

TTV was detected in almost every sample in this study. TTV is a human virus with a circular negative-strand DNA genome of approximately 3.8 kb in length [32, 33]. Although this virus has a high prevalence among the population, its interaction with the host and the etiological relationship with specific diseases are not yet fully understood. There is a significant correlation between TTV load and airflow limitation in the peripheral airways, as well as the severity of bronchiectasis and decreased lung function [34]. Therefore, TTV may cause influenza-like symptoms. However, whether TTV is the cause of influenza-like symptoms, and the reason why TTV resides in certain individuals, even healthy individuals, requires further research.

Several studies have shown that children with MP-related extra-pulmonary diseases higher IgE levels exhibit more severe clinical symptoms and complications. Therefore, it is speculated that IgE might be a biomarker for complications after MP infection [35, 36]. Respiratory virus infection is a further clinical complication for children with MP, and may increase the severity of disease [37]. Although clinical presentation and laboratory tests were not statistically significant between children with single MP infection and children with MP and virus coinfection in this study, the impact of particular virus on MP infection outcomes and the clinical manifestations of MPP require further investigation to find virus-related biomarkers. Therefore, it is recommended that pediatricians consider the co-infection of MP and respiratory viruses in the diagnosis and treatment of respiratory diseases. Medical workers should adhere to strict hand washing procedures to avoid more serious complications in children infected with MP.

Our study has several limitations. First, some important laboratory information such as neutrophils, thrombocytes count, hemoglobin level, lactate dehydrogenase, aspartate transaminase and alanine transaminase are not included and do not provide full definition of study population characteristics. Second, the sample size was limited and all the samples were collected from one sentinel hospital in urban areas of Beijing, our findings may not be representative of viral spectrum in children diagnosed with MPP in China. Finally, we only focused on co-infection with multiple viruses, the co-infection of MP with a specific virus may provide greater insight into the impact of certain virus co-infections on MPP.

## Conclusion

NGS analysis revealed 11 virus families in both MP-positive and MP-negative samples, the highest number of reads was the *Pneumoviridae* and HRSV in MP-positive samples, which was much higher than MP-negative samples. Among MP-positive cases, IFV co-infection were the most cases. By contrast with MP isolated infection children, the clinical symptoms of children with MP combined with virus infection had no statistically difference. Our study provided a theoretical basis for the development of effective prevention and treatment strategies for MPP.

## Supplementary Information

**Additional file 1**. The tables of the filtering parameters setting using Prinseq-lite software, primers and probes used to detect co-infection respiratory viruses, type analysis of common virus-related sequence viral in MP positive and MP negative (TTV has been removed).** Table S1**: The filtering parameters setting using Prinseq-lite software.** Table S2**: Primers and probes used to detect co-infection respiratory viruses.** Table S3**: Type analysis of common virus-related sequence viral in MP positive and MP negative (TTV has been removed).

## Data Availability

The data set supporting the conclusions of this paper is included in the article.

## Abbreviations

CAP: Community-acquired pneumonia
MP: *Mycoplasma pneumoniae*
NPAs: Nasopharyngeal aspirates
MPP: *Mycoplasma pneumoniae* pneumonia
HRSV: Human respiratory syncytial virus
HAdV: Human adenovirus
IFV: Influenza virus
mNGS: Metagenomic next generation sequencing
RTIs: Respiratory tract infections
HPIV: Human parainfluenza virus
HMPV: Human metapneumovirus
HPyV: Human polyomavirus
HRV: Human rhinovirus
HBoV: Human bocavirus
HCoV: Human coronavirus
TTVs: Torque teno virus
CT: Cycle threshold
NGS: Next-generation sequencing
PCR: Polymerase chain reaction
LP: *Legionella pneumophila*
ILS: Influenza-like symptoms.
