# Supplementary material for: Comprehensive virome analysis of the viral spectrum in paediatric patients diagnosed with Mycoplasma pneumoniae pneumonia
Source: Virol J. 2022 Nov 9;19:181. doi: 10.1186/s12985-022-01914-y (PMC9644017; doi:10.1186/s12985-022-01914-y)
Supplement: Supplementary file 1 — Additional file 1. The tables of the filtering parameters setting using Prinseq-lite software, primers and probes used to detect co-infection respiratory viruses, type analysis of common virus-related sequence viral in MP positive and MP negative (TTV has been removed). Table S1: The filtering parameters setting using Prinseq-lite software. Table S2: Primers and probes used to detect co-infection respiratory viruses. Table S3: Type analysis of common virus-related sequence viral in MP positive and MP negative (TTV has been removed). [file 12985_2022_1914_MOESM1_ESM.docx]

**Table S1** The filtering parameters setting using Prinseq-lite software

| **Filter options** | **Value** |
| --- | --- |
| -range_gc | 20-80 |
| -min_qual_score | 22 |
| -lc_method | entropy |
| -lc_threshold | 68 |
| -derep | 1 |
| -ns_max_n | 20 |

**Table S2** Primers and probes used to detect co-infection respiratory viruses

| **Primers** | **Primer sequence (5’-3’)** |
| --- | --- |
| HAdV Forward  HAdV Reverse  HAdV Probe  HPyV 3 Forward  HPyV 3 Reverse  HPyV 3 Probe  HPyV 4 Forward  HPyV 4 Reverse  HPyV 4 Probe  HRSV Forward  HRSV Reverse  HRSV Probe  HMPV Forward  HMPV Reverse  HMPV Probe  HRV Forward  HRV Reverse  HRV Probe  HBoV Forward  HBoV Reverse  HBoV Probe  IFV A Forward  IFV A Reverse  IFV A Probe  IFV B Forward  IFV B Reverse  IFV B Probe  IFV C Forward  IFV C Reverse  IFV C Probe  HPIV 1 Forward  HPIV 1 Reverse  HPIV 1 Probe  HPIV 2 Forward  HPIV 2 Reverse  HPIV 2 Probe  HPIV 3 Forward  HPIV 3 Reverse  HPIV 3 Probe  HPIV 4 Forward  HPIV 4 Reverse  HPIV 4 Probe  HCoV-HKU1 Forward  HCoV-HKU1 Reverse  HCoV-HKU1 Probe  HCoV-229E Forward  HCoV-229E Reverse  HCoV-229E Probe  HCoV-OC43 Forward  HCoV-OC43 Reverse  HCoV-OC43 Probe  HCoV-NL63 Forward  HCoV-NL63 Reverse  HCoV-NL63 Probe | GCCCCAGTGGTCTTACATGCACATC  GCCACGGTGGGGTTTCTAAACTT  FAM-TGCACCAGACCCGGGCTCAGGTACTCCGA-TAMRA  GCTAACAAGGCCAAGAAGTCAAG  GCTAGTACTTCTACCCCTCCTTTTTTT  FAM-GGGGGGGGTACAAACTTCTGGCAACA-TAMRA  CCAATGGTACTGTGCCTCATGT  CCATGATTCAATGCTGTACTTTTCA  FAM-ATTCCAGTTCTGAAACACCCAGGGCAAG-TAMRA  GGCAAATATGGAAACATACGTGAA  TCTTTTTCTAGGACATTGTAYTGAACAG  FAM-CTGTGTATGTGGAGCCTTCGTGAAGCT-TAMRA  CATATAAGCATGCTATATTAAAAGAGTCTC  CCTATTTCTGCAGCATATTTGTAATCAG  FAM-TGYAATGATGAGGGTGTCACTGCGGTTG-TAMRA  TGGACAGGGTGTGAAGAGC  CAAAGTAGTCGGTCCCATCC  FAM-TCCTCCGGCCCCTGAATG-TAMRA  CTGCTGCACTTCCTGATTCAAT  GGAGCTTCTTCCAGAGATGTTC  FAM-ACTGCATCCGGTCTC-TAMRA  GACCRATCCTGTCACCTCTGAC  AGGGCATTYTGGACAAAKCGTCTA  FAM-TGCAGTCCTCGCTCACTGGGCACG-TAMRA  CCCACCRAGCAACAMGG  CCTTCCGACATCAGCTTCACT  FAM-CCCGGAACCCATCCCCGGA-TAMRA  GGCAAGCGACATGCTGAAYA  TCCAGCTGCYTTCATTTGCTTT  FAM-CTCTTCCTTCTGATTTTTTCAAA-TAMRA  GTGATTTAAACCCGGTAATTTCTCA  CCTTGTTCCTGCAGCTATTACAGA  FAM-ACCTATGACATCAACGAC-TAMRA  ATGAAAACCATTTACCTAAGTGATGGA  CCTCCYGGTATRGCAGTGACTGAAC  FAM-TCAATCGCAAAAGC-TAMRA  CCAGGGATATAYTAYAAAGGCAAAA  CCGGGRCACCCAGTTGTG  FAM-TGGRTGTTCAAGACCTCCATAYCCGAGAAA-TAMRA  CAGAYAACATCAATCGCCTTACAAA  TGTACCTATGACTGCCCCAAARA  FAM-CCMATCACAAGCTCAGAAATYCAAAGTCGT-TAMRA  AGTTCCCATTGCTTTCGGAGTA  CCGGCTGTGTCTATACCAATATCC  FAM -CCCCTTCTGAAGCAA-TAMRA  CGCAAGAATTCAGAACCAGAG  GGCAGTCAGGTTCTTCAACAA  FAM–CCACACTTCAATCAAAAGCTCCCAAATG-TAMRA  GCTCAGGAAGGTCTGCTCC  TCCTGCACTAGAGGCTCTGC  FAM –TTCCAGATCTACTTCGCGCACATCC-TAMRA  AGGACCTTAAATTCAGACAACGTTCT  GATTACGTTTGCGATTACCAAGACT  FAM-TAACAGTTTTAGCACCTTCCTTAGCAACCCAAACA-TAMRA |

**Table S3** Type analysis of common virus-related sequence viral in MP positive and MP negative (TTV has been removed)

| **Virus** | **Reads No.（%）** | |
| --- | --- | --- |
|  | **MP（+）** | **MP-free（-）** |
| *Pneumoviridae* | 363111（56.98） | 184083（24.10） |
| Respiratory syncytial virus  Human metapneumovirus | 363111  — | 183895  188 |
| *Picornaviridae* | 127537（20.01） | 170113（22.27） |
| Human rhinovirus  Enterovirus  Human parechovirus  Foot-and-mouth disease virus | 126060  1477  —  — | 168339  1707  60  1 |
| *Polyomaviridae* | 67168（10.54） | 156314（20.46） |
| Human polyomavirus | 67168 | 156314 |
| *Orthomyxoviridae* | 31446（4.93） | 27884（3.65） |
| Influenza virus | 31446 | 27884 |
| *Adenoviridae* | 25080（3.94） | 47860（6.27） |
| Human adenovirus | 25080 | 47860 |
| *Paramyxoviridae* | 14354（2.25） | 80001（11.95） |
| Human respirovirus  Human parainfluenza virus  Human rubulavirus | 4699  9035  620 | 79859  130  12 |
| *Coronaviridae* | 6106（0.96） | 6160（0.81） |
| Human coronavirus | 6106 | 6160 |
| *Parvoviridae* | 2306（0.36） | 91301（11.95） |
| Human bocavirus  Parvovirus  Adeno-associated virus | 989  1119  198 | 91206  15  80 |
| *Caliciviridae* | 115（0.02） | 176（0.02） |
| Norovirus | 115 | 176 |
| *Astroviridae* | 14（<0.01） | 32（<0.01） |
| Mamastrovirus | 14 | 32 |
| *Papillomaviridae* | 11（<0.01） | 2（<0.01） |
| Human papillomavirus | 11 | 2 |
| Total reads | 637248 | 763926 |
